# Regulation of NADPH Oxidase 5 by Protein Kinase C Isoforms

**DOI:** 10.1371/journal.pone.0088405

**Published:** 2014-02-05

**Authors:** Feng Chen, Yanfang Yu, Steven Haigh, John Johnson, Rudolf Lucas, David W. Stepp, David J. R. Fulton

**Affiliations:** 1 Department of Forensic Medicine, Nanjing Medical University, Nanjing, Jiangsu, China; 2 Vascular Biology Center, Georgia Regents University, Augusta, Georgia, United States of America; 3 Department of Pharmacology and Toxicology, Georgia Regents University, Augusta, Georgia, United States of America; Xi'an Jiaotong Univesity School of Medicine, China

## Abstract

NADPH oxidase5 (Nox5) is a novel Nox isoform which has recently been recognized as having important roles in the pathogenesis of coronary artery disease, acute myocardial infarction, fetal ventricular septal defect and cancer. The activity of Nox5 and production of reactive oxygen species is regulated by intracellular calcium levels and phosphorylation. However, the kinases that phosphorylate Nox5 remain poorly understood. Previous studies have shown that the phosphorylation of Nox5 is PKC dependent, but this contention was based on the use of pharmacological inhibitors and the isoforms of PKC involved remain unknown. Thus, the major goals of this study were to determine whether PKC can directly regulate Nox5 phosphorylation and activity, to identify which isoforms are involved in the process, and to understand the functional significance of this pathway in disease. We found that a relatively specific PKCα inhibitor, Ro-32-0432, dose-dependently inhibited PMA-induced superoxide production from Nox5. PMA-stimulated Nox5 activity was significantly reduced in cells with genetic silencing of PKCα and PKCε, enhanced by loss of PKCδ and the silencing of PKCθ expression was without effect. A constitutively active form of PKCα robustly increased basal and PMA-stimulated Nox5 activity and promoted the phosphorylation of Nox5 on Ser490, Thr494, and Ser498. In contrast, constitutively active PKCε potently inhibited both basal and PMA-dependent Nox5 activity. Co-IP and in vitro kinase assay experiments demonstrated that PKCα directly binds to Nox5 and modifies Nox5 phosphorylation and activity. Exposure of endothelial cells to high glucose significantly increased PKCα activation, and enhanced Nox5 derived superoxide in a manner that was in prevented by a PKCα inhibitor, Go 6976. In summary, our study reveals that PKCα is the primary isoform mediating the activation of Nox5 and this maybe of significance in our understanding of the vascular complications of diabetes and other diseases with increased ROS production.

## Introduction

The production of reactive oxygen species (ROS) has been shown to play important roles in both physiological and pathophysiological processes[1]–[4]. Under physiological conditions, ROS production is tightly regulated to release the appropriate amount at the right time and place to influence cellular processes such as intracellular signaling, gene expression, proliferation, migration, differentiation, and hormone synthesis[1], [5], [6]. However, the excessive production of ROS has been shown to contribute to the pathogenesis of cardiovascular diseases, including, diabetes, obesity, atherosclerosis, and systemic and pulmonary hypertension as well as cancer and inflammation[7]–[13].

The scavenging of ROS by non-selective antioxidant therapies has been documented to ameliorate cardiovascular disease in a number of animal models. In humans, however, antioxidants have not been shown to provide significant clinical benefit[14]. Many explanations have been provided to account for the clinical failure of antioxidants including a lack of selectivity in inhibiting both physiological and pathophysiological ROS. Selectively targeting individual ROS generating enzymes, particularly those that are upregulated or hyperstimulated in disease, is likely to be a more effective strategy. However, this approach is currently limited by an incomplete understanding of the molecular regulation underlying ROS production. Towards that end, a better understanding of the mechanism controlling ROS production in disease will aid in the development of more effective therapeutics.

In blood vessels, the majority of ROS derive from a unique family of enzymes that have the unique ability of efficiently synthesizing superoxide from NADPH[15]. There are seven related Nox genes that include Nox1-5 and the Duoxes1 and 2. Vascular cells express Nox1, 2, 4 and 5[16]–[20]. Nox5 was the last Nox enzyme discovered and its activity is regulated by the level of intracellular calcium, and phosphorylation of serine/threonine residues of Ser475, Ser490, Thr494 and Ser498[3], [4], [21]–[23]. The phosphorylation of Nox5 enhances its sensitivity to calcium and enables ROS production at lower levels of calcium[21], [24]. Although originally discovered in testis, lymph nodes, and spleen, Nox5 has been recently shown in blood vessels and the heart, and is expressed in endothelial cells, smooth muscle cells, and primary cardiac fibroblasts[22], [23], [25], [26]. The expression and activity of Nox5 are dramatically elevated in atherosclerosis[24], acute myocardial infarction[27], and fetal ventricular septal defect [28], which suggests that the dysregulation of Nox5 could contribute to cardiovascular disease in humans[25].

Protein kinase C (PKC) refers to a family of related kinases that belongs to the AGC (cAMP-dependent protein kinase/protein kinase G/protein kinase C) superfamily. PKCs are serine/threonine protein kinases that play important roles in signal transduction in health and disease, contributing to endothelial dysfunction, vascular permeability, angiogenesis, cell growth and apoptosis, and extracellular matrix expansion[29]. There are multiple PKC isoforms that participate in a wide variety of biological functions[30]. Previous studies have shown that PKC mediates the phosphorylation of Nox5, but this was based exclusively on the use of pharmacological inhibitors, and the PKC isoforms involved remain to be elucidated [21], [26]. In the current study, we found PKCα directly modifies Nox5 phosphorylation and activity using both pharmacological and genetic approaches, while PKCε and PKCδ influence Nox5-derived superoxide through indirect mechanisms.

Hyperglycemia is a major risk factor for diabetics and has been shown to aggressively increase the severity of atherosclerosis, and microvascular pathologies[29], [31], [32]. In endothelial cells, high glucose induces the activation and translocation of PKCα to the plasma membrane, which results in endothelium-dependent vasodilator dysfunction by altering the bioavailability of nitric oxide (NO) secondary to increased superoxide production from Nox enzymes, and reduced NO production from eNOS [10]. The overproduction of ROS can also reciprocate and activate PKC enzymes and this positive feedback pathway can contribute substantially to diabetic vascular damage[33]. Whether high glucose can contribute to the activation of Nox5 was a goal of the current study and this pathway may have important implications in the development of diabetic vascular complications.

## Materials and Methods

### Cell culture

COS-7 [34]–[36] and HEK cells [35], [37] were cultured in Dulbecco's modified Eagle's medium (Invitrogen, Carlsbad, CA) containing L-glutamine, penicillin, streptomycin, and 10% (v/v) fetal bovine serum. Cells were transfected using Lipofectamine 2000 reagent (Invitrogen) as described previously[34]–[36], [38], [39]. The HA-Nox5 HEK293 cell line was generated by using Flp Recombinase-Mediated Integration (Invitrogen)[36], [37]. Human lung microvascular endothelial cells (HLMVEC) were purchased from Lonza, and were grown in Endothelial Growth Medium-2-Microvessel (EGM-2MV) consisting of defined growth factors and supplemented with additional FBS up to 5% final concentration (Lonza). Cells were grown at 37°C in 5% CO_2_ incubator and used from passage 2–6.

Ro 32-0432 (Bisindolylmaleimide XI hydrochloride) and Gö 6976 were obtained from Sigma-Aldrich (St.Louis, MO). L-glucose and D-glucose were purchased from Thermo Fisher Scientific (Waltham, MA).

### DNA and adenoviral constructs

Plasmid DNA encoding Nox5β (AF325189) has been described previously[34], [39].The Nox5 S490A/T494A/S498A mutant was generated by multiple mutagenesis as previously described[21]. Myr-PKCα, myr-PKCε and PKCε(A159E) were generated by PCR. All constructs were verified by bidirectional sequencing. Control (RFP) and HA-Nox5 adenoviruses have also been described[38], [39].

### Co-immunoprecipitation and Western blotting analysis

Cells were lysed on ice in 20 mM Tris-HCl (pH 7.4), 1% Triton X-100, 100 mM NaCl, 1 mM Na_3_VO_4_, 10 mM NaF, and 1% protease inhibitor cocktail (Sigma). Soluble extracts were incubated for 2 h at 4°C with relevant antibodies: anti-HA (Roche Applied Science) and a negative isotype control mouse immunoglobulin (IgG) (Santa Cruz Biotechnology), and complexes precipitated with protein A/G agarose (Santa Cruz Biotechnology). Western blotting was performed as described previously[22], [40]–[43] using anti-HA (Roche), anti-V5 (Invitrogen), anti-PKCα, β, γ, ε, η, θ, ι, λ and δ (Cell Signaling Technology), anti-ERK1/2 (Cell Signaling Technology), anti-ERK1/2 phosphorylation (Cell Signaling Technology),anti-MEK(Cell Signaling Technology), and anti-GAPDH (Santa Cruz Biotechnology), and anti-Nox5 phosphorylation antibodies[21].

### In Vitro Kinase Assay

Nox5 was purified by immunoprecipitation from COS-7 cells transduced with HA-Nox5 adenovirus and incubated with 100 ng of active PKCα (Life Technologies, Grand Island, NY) for 30 min at 30°C in kinase buffer containing 20 mM HEPES, pH 7.4, 10 mM MgCl2, 100 µM CaCl2, 100 µg/ml phosphatidylserine, 0.03% Triton X-100, with or without 100 µM ATP. The reaction was terminated by the addition of SDS sample buffer. Incorporation of phosphate into Nox5 was determined using by SDS-PAGE followed by immunoblotting using phosphorylation state-specific antibodies that recognize phosphorylated Nox5 at Ser490, Thr494, and Ser498.

### Transient knockdown of PKC gene with siRNA

The siRNA targeting PKCα (siRNA ID: s11094), PKCε (siRNA ID: s11101), PKCδ (siRNA ID: s11099) and PKCθ (siRNA ID: s11122) were obtained from Applied Biosystems. Validated control and targeting siRNA were transfected into HEK293 cells stably expressing Nox5 using siPORT™ Amine (Applied Biosystems).

### Measurement of Superoxide

Cells were plated into white tissue culture treated 96-well plates (Thermo Fisher Scientific) at a density of approximately 5×10^4^cells/well. The cells were incubated at 37°C in phenol-free Dulbecco's modified Eagle's medium (Sigma-Aldrich, St. Louis, MO) containing 400 µM concentration of the luminol analog 8-amino-5-chloro-7-phenylpyrido[3,4-d]pyridazine-1,4-(2H,3H) dione (L-012) (Wako Pure Chemicals, Tokyo, Japan) for a minimum of 20 min before the addition of agonists. Luminescence was quantified over time using a Lumistar Galaxy (BMG Labtech, Durham, NC) luminometer as described [21], [34]–[37], [39]. The specificity of L-012 for superoxide was confirmed by transfecting cells with a control plasmid such as green fluorescent protein or lacZ or by co-incubation of a superoxide scavenger such as Tiron (5 mM). Both of these interventions yielded virtually undetectable levels of luminescence under control, PMA-, ionomycin- or PLY-stimulated conditions. Superoxide production is recorded as relative light units (RLU) and as such, the absolute levels of ROS in separate experiments are not directly comparable.

### Statistical Analysis

Data were reported as mean ± SE and statistical analyses were performed using Instat software (GraphPad Software Inc., San Diego, CA) with a two-tailed student's t-test or ANOVA with a post-hoc test where appropriate. Differences were considered as significant at p<0.05.

## Results

### Dose-dependent inhibition of Nox5 activity by conventional PKC inhibitors and the calcium-dependency of PMA induced Nox5 phosphorylation

**Figure 1:**
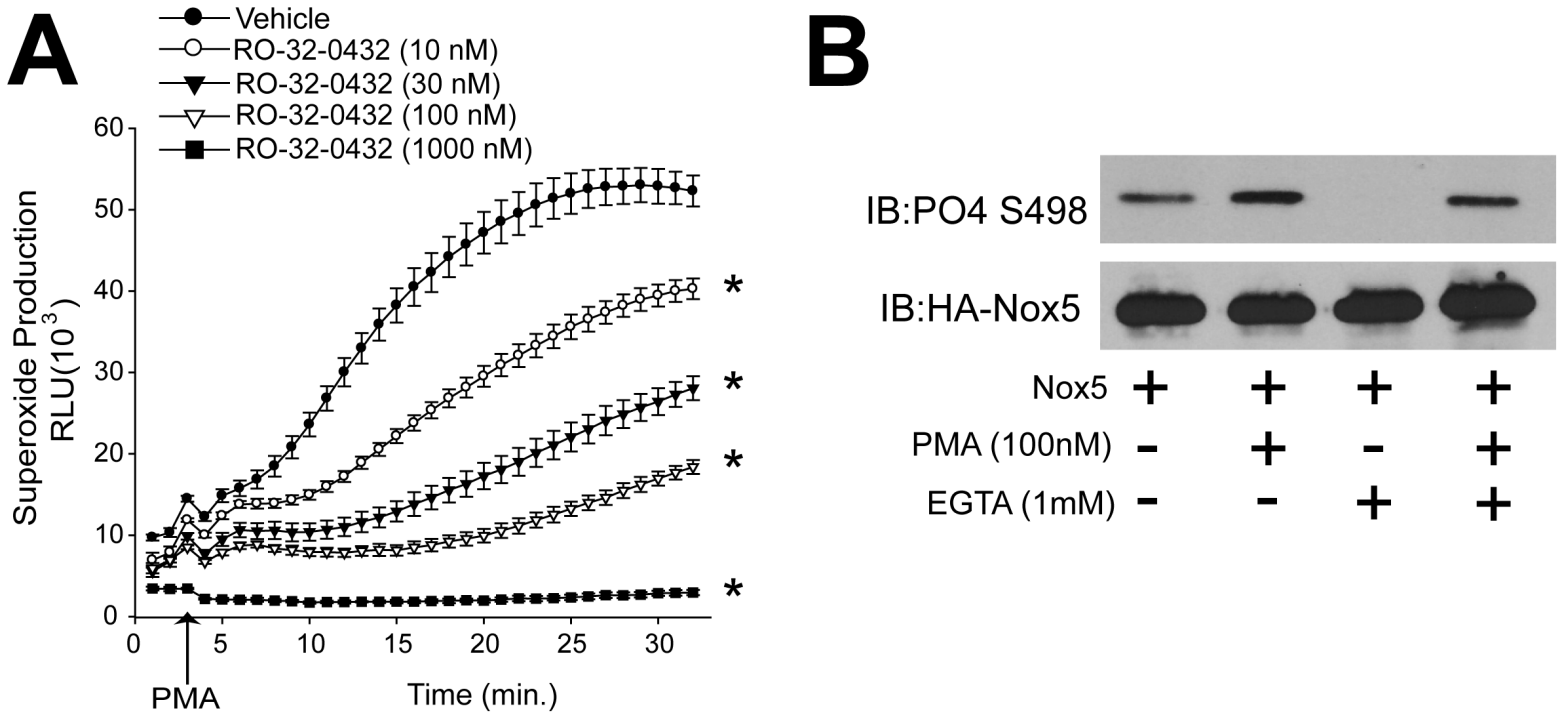
Dose-dependent inhibition of Nox5 activity by a PKCα selective inhibitor and the calcium-dependency of PMA induced Nox5 phosphorylation. (A) COS-7 cells expressing Nox5 were pretreated with vehicle (DMSO) or different doses of RO-23-0432 (10 nM-1000 nM) for 1 hour prior to stimulation with PMA (100 nM), and superoxide production was measured using L-012 chemiluminescence over time as indicated (means ± S.E., n = 5). (B) The ability of EGTA (1 mM) to modulate PMA-dependent phosphorylation of Ser498 was determined by immunoblotting (IB) (top) relative to the level of total Nox5 protein (bottom). Results are representative of at least 3-5 separate experiments, presented as means ± S.E., * p<0.05 versus Vehicle.

Our previous study reported that the protein kinase C(PKC)-agonist PMA could induce a sustained activation of Nox5, and the conventional PKC inhibitors, rottlerin and LY379196, reduced Nox5 activity[21] In this study, to further test whether PKCs and determine PKC isoforms are involved in PMA-dependent activation of Nox5, COS-7 cells expressing Nox5 were incubated with a relative selective PKCα inhibitor, RO-32-0432 (Figure 1A). As we can see, RO-32-0432 significantly attenuated PMA-dependent increases in Nox5 activity and Nox5 phosphorylation on Ser498, suggesting that PKC isoforms participate in the activation and phosphorylation of Nox5. The PKC family encompases more than 10 different isoforms, and these can be subclassified into calcium sensitive and insensitive isoforms. To determine whether calcium is important for Nox5 phosphorylation in response to PMA, we treated cells expressing Nox5 with the calcium chelator EGTA. EGTA dramatically reduced the basal phosphorylation of Nox5 on Ser498 and reduced PMA-dependent phosphorylation. This data indicates that calcium is necessary for PMA induced Nox5 phosphorylation (Figure 1B).

### PKCα directly modifies Nox5 phosphorylation and activity

To examine which PKC isoform participates in Nox5 phosphorylation and activity, we first obtained the expression profile of PKC isoforms in COS-7 cells using Western blot. We found that PKCα, ε, θ, ι, λ and δ are the predominant PKC isoforms in COS-7 cells. Of these, PKCα, ι, λ and δ appear to be the ones most strongly expressed (Table 1 and Supplemental Figure 1).

**Table 1:** Properties and relative protein expression of PKC isoforms in COS-7 cells.

PKC ISOFORMS	PMA dependent	Ca2+ dependent	Expressed in COS cells
*Alpha*	*YES*	*YES*	*+++*
*Beta I*	*YES*	*YES*	*+*
*Beta II*	*YES*	*YES*	*+*
*Delta*	*YES*	*NO*	*+*
*Eta*	*YES*	*NO*	*−*
*Epsilon*	*YES*	*NO*	*+*
*Gamma*	*YES*	*YES*	*−*
*Iota*	*NO*	*NO*	*++*
*Lamda*	*NO*	*NO*	*++*
*Theta*	*YES*	*NO*	*+*
*Zeta*	*NO*	*NO*	*++++*

**Figure 2:**
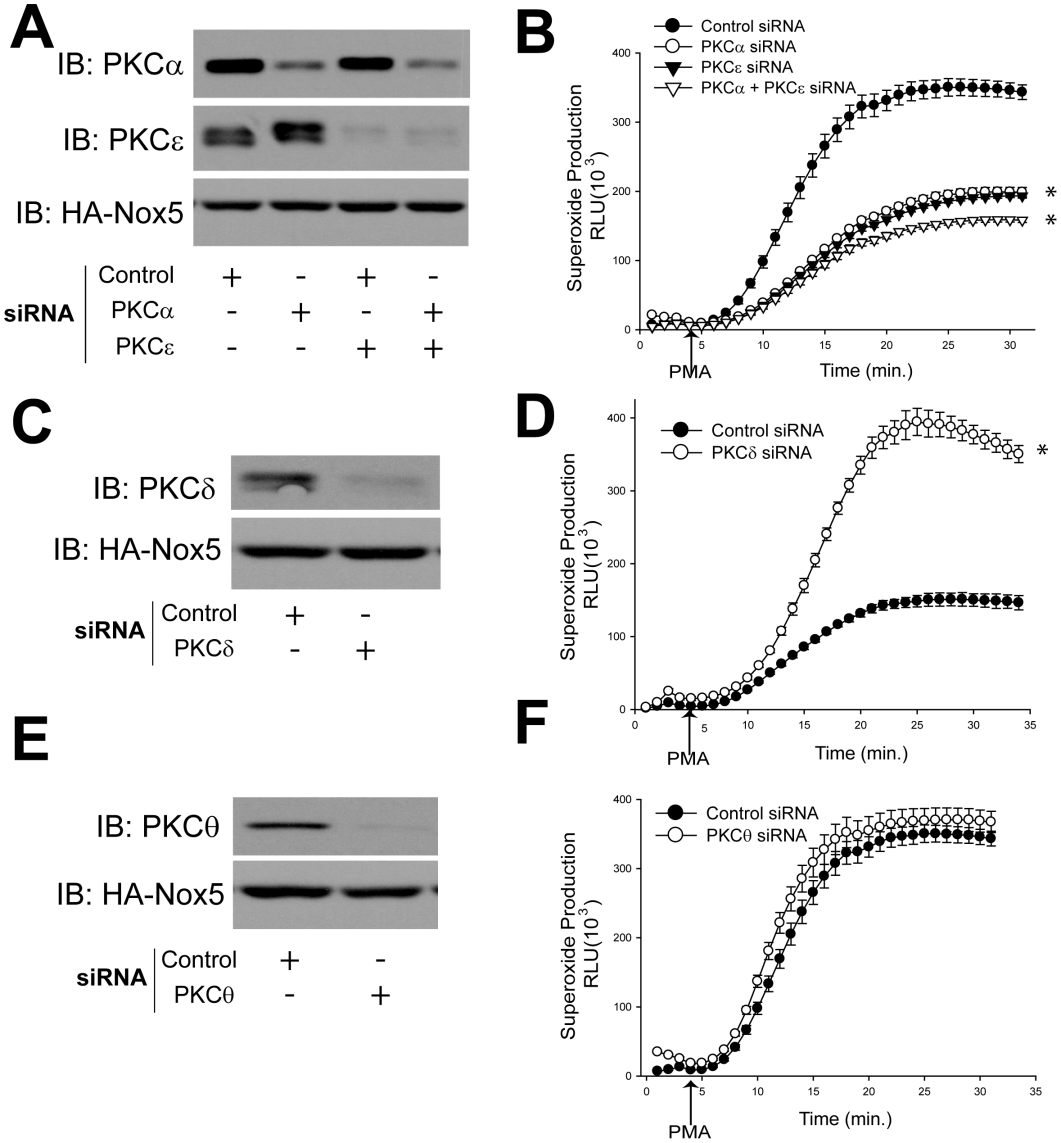
Silencing PKCα and ε, but not PKC δ and θ, by siRNA reduces Nox5 derived superoxide production. HEK cells stably expressing Nox5 were transfected with negative control siRNA or siRNA targeting PKCα, ε, δ, θ for 48 h. Lysates were immunoblotted for PKCα, ε, δ, θ, Nox5 and GAPDH as loading control (A, C, E). Superoxide production was measured using L-012 chemiluminescence in response to PMA (100 nM) stimulation (B, D, F) (means ± S.E., n = 6). Results are representative of at least 3–5 separate experiments, presented as means ± S.E., * p<0.05 versus Vehicle.

The activation mechanisms of each of the PKC isoforms are different: the conventional PKC isoforms (PKCα, β1, β2, and γ) are activated by calcium and DAG or phorbol esters such as phorbol 12-myristate 13-acetate (PMA), and phosphatidylserine (PS), whereas novel PKCs (PKCδ, ε, θ, and η) are activated by DAG or mimetics such as PMA, PS, but not by calcium. The atypical PKCs (PKCζ and ι/λ) are not activated by calcium, DAG or PMA. Based on the expression profile and PKC activation properties, we hypothesized that PMA-dependent Nox5 activity is likely to be regulated by PKCα, ε, δ, and θ isoforms. To determine whether endogenous PKC isoforms are necessary for Nox5 activity in response to PMA, we silenced PKCα, ε, δ and θ using a siRNA based approach. In cells with diminished levels of PKCα or PKCε there was a robust reduction on the level of superoxide production from Nox5 (Figure 2A-B), however, Nox5 derived superoxide production was only slightly reduced with the combination of PKCα and PKCε siRNA compared to PKCα or PKCε alone.

While loss expression of PKCδ significantly increased Nox5 derived superoxide (Figure 2C–D). Silencing of PKCθ did not have an effect on Nox5 activity (Figure 2E–F).

**Figure 3:**
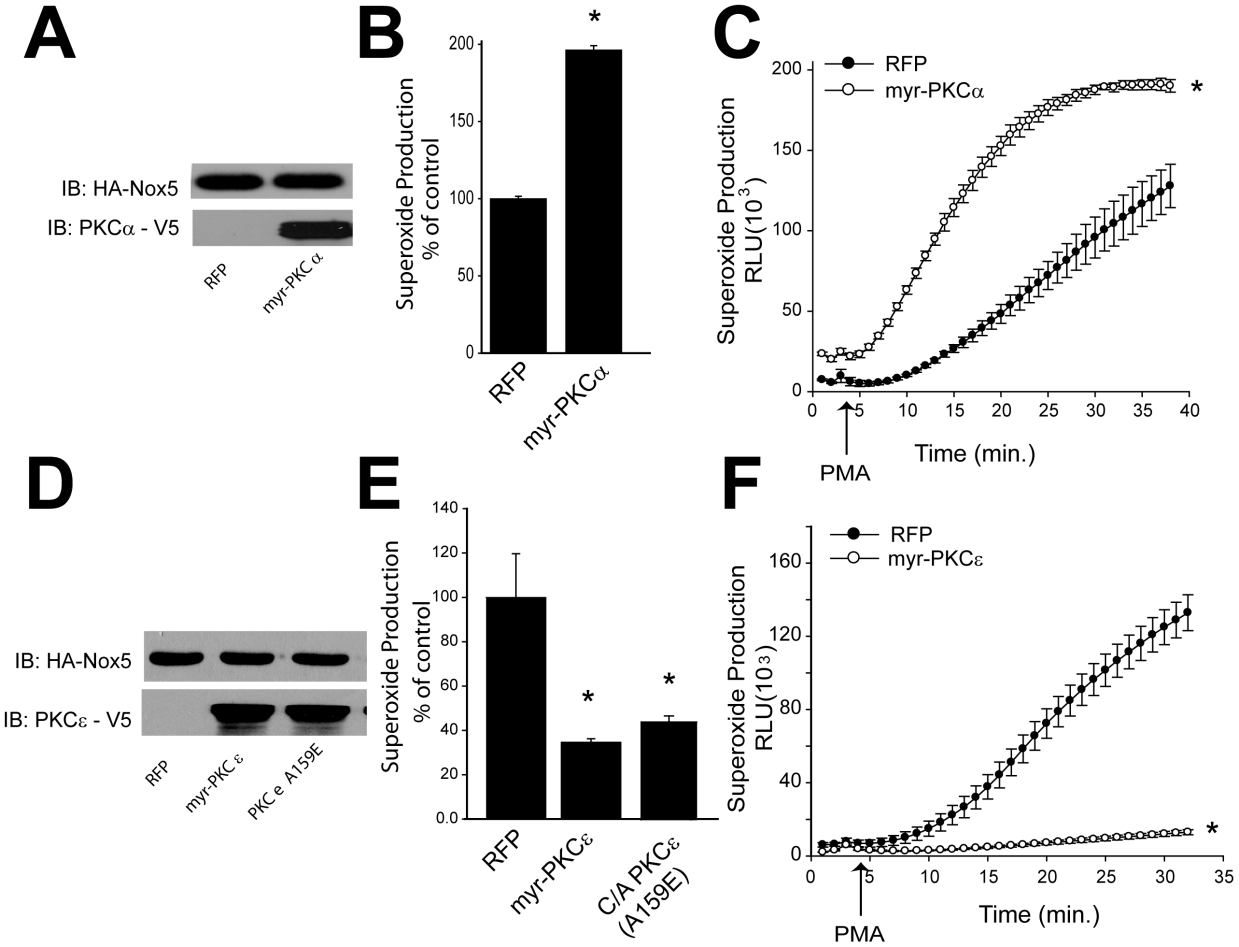
A constitutively active form of PKCα increases basal and stimulated Nox5 activity. HEK cells stably expressing Nox5 were transfected with either control plasmid (RFP) or myr-PKCα (A), myr-PKCε, PKCε A159E (D), and cell lysates were immunoblotted with V5 and HA antibodies. Basal (B, E) and PMA stimulated (C, F) superoxide production were measured using L-012 chemiluminescence. (means ± S.E., n = 6–8). Results are representative of at least 3 separate experiments, presented as means ± S.E., * p<0.05 versus Vehicle.

As these results support the importance of PKCα and ε in regulating Nox5 activity, our next goal was to see whether active forms of PKCα and ε are can stimulate increased Nox5 activity. We transfected HEK cells stably expressing Nox5 with constitutively active forms of PKCα or ε (myr-PKCα or ε), and measured Nox5-dependent superoxide production. As shown in Figure 3A–C, we observed a robust increase in Nox5 activity in cells expressing myr-PKCα under both basal and PMA stimulated conditions. However, myr-PKCε significantly reduced Nox5-dependent superoxide production (Figure 3D–F). This result was contrary to expectations and we repeated this experiment with a different type of constitutively active PKCε (PKCε A159E), which yielded the same result (Figure 3D–F).

**Figure 4:**
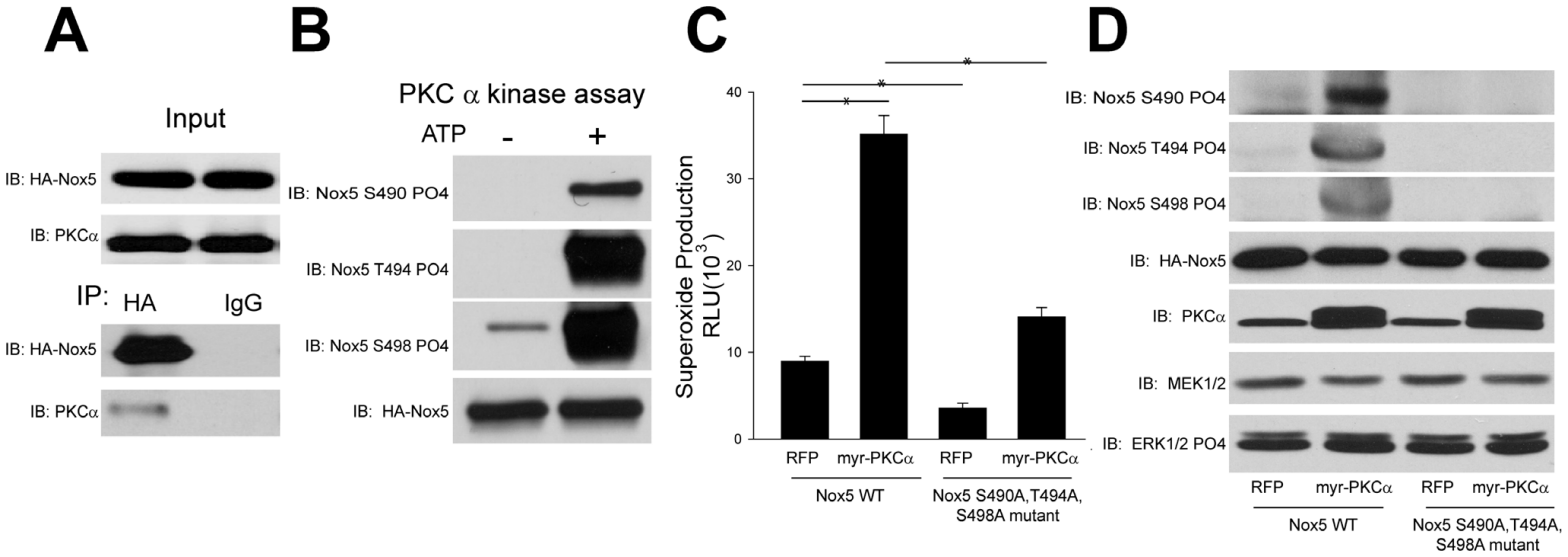
PKCα binds to Nox5 and regulates its activity through direct phosphorylation of Ser490, Thr494 and Ser498. (A) COS-7 cells expressing Nox5 were lysed and immunoprecipitated using either control IgG or anti-HA antibody. Immune complexes were immunoblotted for HA-Nox5 or PKCα. (B) Nox5 was immunoprecipitated from COS-7 cells transduced with HA-Nox5 adenovirus and subject to an in vitro kinase assay. Phosphorylated samples were immunoblotted for phosphorylated Ser490, Thr494, and Ser498 versus total Nox5. Superoxide release from COS-7 cells cotransfected with HA-Nox5 WT or triple mutant (Nox5 S490A,T494A,S498A) and either control (RFP) or myr-PKCα (C). Lysates were immunoblotted for PKCα, MEK1/2, ERK1/2 phosphorylation, phosphorylated S490, T494 and S498 relative to total Nox5 (HA) expression (D). Results are representative of at least 3 separate experiments, presented as means ± S.E., * p<0.05 versus Vehicle.

To determine whether PKCα can modify Nox5 activity by direct binding and site-specific phosphorylation, we next conducted a co-immunoprecipitation experiment. We found evidence for a strong physical association between Nox5 and PKCα (Figure 4A). To determine whether PKCα can directly phosphorylate Nox5, we performed an *in vitro* kinase assay using immunoprecipitated Nox5 as a substrate. As shown in Fig. 4B, we found that active PKCα robustly increased the phosphorylation of Nox5 on Ser490, Ser494, and Thr498 in the presence of ATP. Together, these data strongly suggest that PKCα can directly modify Nox5 phosphorylation and activity through direct binding to the enzyme.

### PKCα increased Nox5 phosphorylation at the sites of Ser490, Thr494 and Ser498

Using a site-specific mutagenesis approach, our previous study had identified three Nox5 phosphorylation sites, Ser490, Thr494 and Ser498, which are phosphorylated to different degrees by PMA. It is not yet known whether these Nox5 phosphorylation sites are regulated by PKCα and to test this we used phosphorylation state-specific antibodies to Ser490, Thr494 and Ser498. We found that PKCα significantly increased Nox5 activity and phosphorylation at sites of Ser490, Thr494 and Ser498 without modifying the MAPK pathway (Figure 4C–D). The ability of PKCα to stimulate Nox5 activity was significantly reduced in the Nox5 triple mutant (Nox5 S490A, T494A, S498A) and site-dependent phosphorylation of S490, T494 and S498 absent (Fig.4C–D). However, the activity of the Nox5 triple mutant was also increased above baseline by PKCα, suggesting the other kinases may also be involved in the process of Nox5 phosphorylation at other sites, such as Ser475 or other pathways of activation[3].

### The PKCα pathway contributes hyperglycemia induced Nox5 hyperactivity

**Figure 5:**
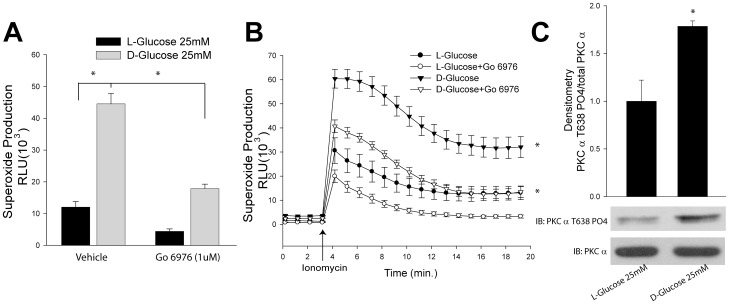
High glucose increases Nox5 derived superoxide production by activating the PKCα pathway. COS-7 cells expressing Nox5 were exposed L-Glucose (25 mM) or D-glucose (25 mM) for 6 hours, and then treated with vehicle (DMSO) or PKCα inhibitor, Go6976. Superoxide production was measured under basal (A) and ionomycin-stimulated conditions (0.4 µM) (B). (C) COS-7 cells were treated with either L-Glucose (25 mM) or D-glucose (25 mM) for 6 hrs, and membrane faction which was purified from total cell lysate was immunoblotted with PKCα Thr 638 phosphorylation antibody and total PKCα antibody. Results are presented as means ± S.E., n = 6, * p<0.05 versus Vehicle.

To explore the significance of this pathway in diabetes, we next measured superoxide production in COS-7 cells expressing Nox5 exposed to high glucose (D-Glucose, 25 mM) or osmotic control (L-Glucose, 25 mM) in the presence and absence of a more selective PKCα inhibitor, Go 6976. We found that high glucose significantly increased Nox5 activity, and the inhibition of PKCα reduced both basal and stimulated superoxide production from Nox5 (Figure 5A–B). This effect is correlated with increased PKCα activation as evidenced by phosphorylation at Thr638 (Figure 5C). In HLMVEC cells, we also found high glucose significantly increased Nox5 activity in response to the gram positive toxin, PLY (Supplemental Figure 2A). To confirm the source of superoxide in Nox5-transduced HLMVECs, cells were transduced with either RFP or Nox5 adenovirus. As shown in Supplemental Figure 2B, superoxide production was only detected in cells transduced with Nox5 virus and not with the control virus, RFP.

## Discussion

Previous studies have shown that the PKC activator, PMA, exhibits a robust stimulation of superoxide from Nox5 without changing the levels of intracellular calcium, an effect dependent on the phosphorylation of Nox5 on Thr494 and Ser498 [4], [21]. Although PMA is considered a PKC agonist, studies have shown that PMA can also activate other kinases including members of the mitogen-activated protein kinase 1 and 2 (MAPK) pathway[4]. Previously, we have also reported that PMA can stimulate ERK1/2 phosphorylation and that ERK can directly influence Nox5 activity through the phosphorylation of a distinct serine residue. Accordingly, it remains unknown whether PKCs can directly regulate Nox5 phosphorylation and if so, which isoforms of PKC mediate this effect. By using a complementary pharmacological and genetic approach, we have found evidence that primarily supports a role for PKCα. A selective inhibitor of PKCα and conventional PKC isoforms, Ro-32-0432, dose dependently inhibited superoxide production from Nox5. A role of PKCα is further supported by the reduced Nox5 activity observed in cells where PKCα has been silenced by siRNA. Gain of function strategies also support a role for PKCα in that a constitutively active form of PKCα (myr-PKCα) robustly increased Nox5 activity and promoted the phosphorylation of Nox5 on Ser490, Thr494, and Ser498. Mutation of these sites to non-phosphorylatable alanine residues blunts the ability of PKCα to stimulate superoxide release from Nox5. Co-IP experiments reveal that PKCα binds directly to Nox5 and to determine whether PKCα can function as the terminal kinase that directly phosphorylates Nox5, we performed an *in vitro* kinase assay. We found active recombinant PKCα robustly increased Nox5 phosphorylation on Ser490, Ser494, and Thr498 in the presence of ATP. Basal phosphorylation in the absence of ATP was minimal. This suggests that indeed, PKCα can bind to and directly phosphorylate Nox5. Collectively, these results suggest that PKCα is the premier PKC isoform regulating Nox5 activity through the direct phosphorylation of Ser490, Thr494 and Ser498. However, our data also suggests that PKCα is clearly not the only kinase involved.

Any study of PKC-dependent events is complicated by the simultaneous presence of multiple isoforms of PKCs. The expression profile of PKC isoforms varies depending on the cell type, tissue and experimental conditions. Our study revealed that PKCα, ε, θ, ι, λ, and δ are the most abundant isoforms expressed in COS-7 cells using Western blotting, however, due to the limitation of Western blotting using different antibodies and exposure times, the exact protein expression profile remains uncertain. This could be addressed by two-dimensional gel electrophoresis and liquid chromatography tandem mass spectrometry (2D LC-MS/MS) but is beyond the scope of the present study [44], [45]. The rigor of a pharmacological approach can be improved using inhibitors with common molecular targets and disparate chemical structures. Initial screening experiments used Ro 32-0432 (Fig.1) which inhibits conventional PKC isoforms with limited selectivity (binding affinities for PKCα, βΙ, βΙΙ, γ and ε are 9, 28, 31, 37 and 108 nM, respectively). In subsequent studies, with knowledge of PKCα involvement, we also used Gö 6976 (Fig.5) which is a potent and selective PKCα inhibitor (IC_50_ = 2.3 nM), but does not inhibit the activity of PKCδ, −ε, or −ζ. A role for PKCα is further supported by complementary loss and gain of function genetic approaches.

The fact that loss expression of PKCδ significantly increased Nox5 derived superoxide was unexpected and suggests that PKCδ may repress the activity of other kinases or promote lower levels of intracellular calcium which is the primary determinant of Nox5 activity. Others have shown that PKCδ can regulate Nox1 expression and activity[46], [47] and the phosphorylation of p47phox[48] but loss of PKCδ clearly has an overall negative role in Nox5 activity in COS-7 cells. Silencing PKCε also robustly inhibited the PMA-dependent activation of Nox5, an effect equal to that of PKCα. Interestingly, silencing of both α and ε PKC isoforms yielded a combined effect that was only marginally more effective than either isoform alone. These results suggest a degree of interoperability between these isoforms and evidence for cooperation between α and ε isoforms has previously been demonstrated in the activation of other kinase substrates[49], [50]. An unexpected observation was the ability of the constitutively active forms of PKCε (myr- PKCε and PKCε A159E) to significantly reduce Nox5-dependent superoxide production. This suggests that the net ability of PKCδ and PKCε to modify Nox5 activity is probably through an indirect mechanism by regulating a secondary molecule or kinase which might be important for Nox5 activity. Both PKCα and ε have been shown to activate other kinases and we have previously shown that ERK can phosphorylate and activate Nox5. However in the context of the current study, expression of a constitutively active PKCα did not increase ERK phosphorylation suggesting this pathway is not involved. PKCα has also been shown to activate Akt[51], however, whether AKT can regulate Nox5 activity is still unknown. While the overall mechanisms by which PKCδ and PKCε regulate the release of superoxide from Nox5 remain to be determined, the evidence for a role of PKCα is substantial. Not only does PKCα bind directly, but both loss of function and gain of function studies show a major functional effect.

Nox5 has gained significance in recent times with numerous studies revealing it to be an important regulator of cell behavior, including cell growth, differentiation and migration. The overproduction of ROS from Nox5 is thought to contribute to human disease, such as human coronary artery disease[24], atherosclerosis[24], acute myocardial infarction[27], fetal ventricular septal defect[28], and cancer[52]–[56]. Unlike other Nox enzymes, Nox5 is a calcium-dependent enzyme and functions independent of the cytosolic and transmembrane subunits including p40, p47, p67 and p22phox. PKCs have been shown to regulate Nox activity by phosphorylation of these subunits[57]–[65] and there is also some evidence for the direct phosphorylation of Nox1, Nox2 and Nox4[66]–[68]. PKCs have also been shown to induce the expression of Nox1 and Nox4 under different conditions [30], [47], [69], [70]. Similar to Nox5, studies have also shown that the PKCα isoform is responsible for stimulus-driven ROS production from Nox2[71]. Nox5 has also been shown to be activated by other kinases including c-Abl[72], camkII[3] and MAP kinase[4]. Our study, adds PKCα to this list.

Our group has previously found that Nox5 expression and activity are regulated by protein-protein interaction with the molecular chaperones, Hsp90 and Hsp70[35], [36], S-nitrosylation[37] and possibly Sumoylation[39]. How the PKCα-dependent phosphorylation of Nox5 integrates with those mechanisms of activation are not yet known and is a subject that may warrant further investigation. A close relationship exists between ROS and PKC signaling, and elevated ROS, particularly in the form of hydrogen peroxide, can promote increased PKC activity[33]. In the present study, we found that PKCα activation increased ROS production from Nox5. Elevated ROS may then increase PKCα activity. This pathway may act as a positive feedback loop to inappropriately keep Nox5 activity elevated in disease states.

A study by Liu et al. found that PKCα knockout mice exhibit increased myocyte contractility, and are less susceptible to heart failure. In contrast, PKCβγ knockout mice have the same susceptibility as wild type mice, which suggests that the PKCα isoform is the primary regulator of cardiac contractility and susceptibility to heart failure[73]. Administration of specific PKCα/β/γ inhibitors, Ro-32-0432, Ro-31-8220 orruboxistaurin (LY333531), can protect against heart failure in wide type mice, but not in PKCα knockout mice. More importantly, PKCα protein levels and activity are significantly upregulated in both human and experimental models of heart failure[74]–[77]. However, the exact mechanisms underlying the protective effect of PKCα inhibition in heart failure remain elusive. Nox5 has been recently identified in intramyocardial blood vessels and cardiomyocytes after acute myocardial infarction, as well as coronary artery disease in human[24], [27] and is an important modulator of vascular function[22], [78]. Our study reveals an ability of PKCα to directly interact with Nox5 and regulate its activity which may provide as an important mechanism by which inhibition of PKCα protects against cardiovascular disease, including heart failure, myocardial infarction, and coronary artery diseases.

The cardiovascular complications of diabetes are a major cause of suffering in diabetic patients, and hyperglycemia is a major systemic risk factor for endothelial and other vascular dysfunctions[29]. Exposure to high glucose induces the membrane translocation and activation of PKCα [29]. Activation of PKCα reduces the bioavailability of endothelium-derived NO by increasing superoxide production from NADPH oxidase[29]. The pore-forming virulence factor pneumolysin (PLY) released from S. pneumoniae in patients with pneumonia is a major factor responsible for the induction of acute lung injury, especially after aggressive antibiotic therapy which promotes the release of PLY from bacteria [79]. The instillation of purified PLY into murine lungs promotes injury and microvascular barrier disruption that replicates that seen in pneumonia. PLY, alters cell signaling via calcium entry followed by calcium dependent activation of the PKCα isoform through toxin-induced pores. These events disrupt the endothelial cell barrier and can induce endothelial apoptosis as well as increase pro-inflammatory cytokines and chemokines[80]. In the present study, we found that exposure of cells to high glucose significantly increased PKCα activation as determined by Thr638 phosphorylation, and enhanced Nox5 derived superoxide production in both COS-7 cells and HLMVEC in a PKC-dependent manner. Upon stimulation with PLY, we detected a burst of Nox5-derived superoxide production in HLMVEC at 1 min and this acute effect of PLY is mediated by increased intracellular calcium. Over a longer time period (with 30 mins), PLY can cause the activation of PKCα which will further stimulate Nox5 activity and lead to a more significant release of oxidants. The ability of PKCα to stimulate Nox5 might have important implications in the treatment of diabetic vascular complications as well as acute long injury.

In summary, we have found that PKCα is a direct regulator of Nox5 phosphorylation and activity using both pharmacological and gain and loss of functions genetic approaches. Exposure of endothelial cells to high glucose significantly increased PKCα activation, and enhanced Nox5 derived superoxide production which can be prevented by a PKCα inhibitor. This pathway may be of importance in the treatment of cardiovascular diseases, including heart failure, myocardial infarction, and coronary artery disease, particularly in the setting of diabetes and acute lung injury.

## Supporting Information

Figure S1 **Properties and relative protein expression of PKC isoforms in COS-7 cells.** The relative expression level of PKC isoforms in COS-7 cells was determined by immunoblotting with PKCα, β, γ, ε, η, θ, ι, λ and δ antibodies. Results are representative of at least 3–5 separate experiments.
(TIF)

Figure S2 **High glucose increases Nox5 derived superoxide production in HLMVEC in response to PLY.** (A) HLMVC cells were infected with Nox5 adenovirus (20 MOI) for 48 hrs, and then treated with L-Glucose (25 mM) or D-glucose (25 mM) for 6 hours. Superoxide production was measured in response to PLY (60 ng/ml). Results are presented as means ± S.E., n = 6, * p<0.05 versus L-Glucose. (B) HLMVC cells were infected with RFP or Nox5 adenovirus (20 MOI) for 48 hrs, and superoxide production was measured using L-012 chemiluminescence.
(TIF)
