# Supplementary figures and images for: Regulation of NADPH Oxidase 5 by Protein Kinase C Isoforms
Source: PLoS One. 2014 Feb 5;9(2):e88405. doi: 10.1371/journal.pone.0088405 (PMC3914983; doi:10.1371/journal.pone.0088405)

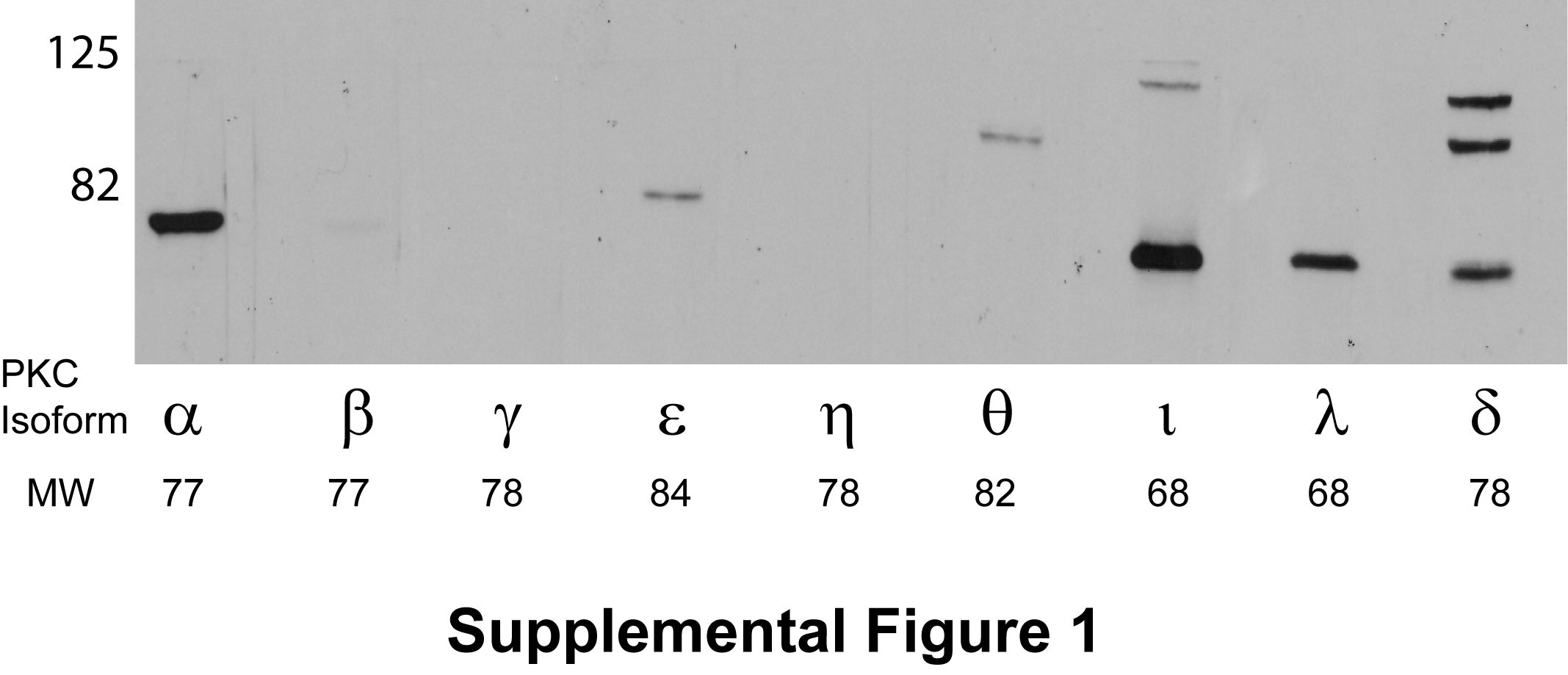

Supplement: Figure S1 — Properties and relative protein expression of PKC isoforms in COS-7 cells. The relative expression level of PKC isoforms in COS-7 cells was determined by immunoblotting with PKCα, β, γ, ε, η, θ, ι, λ and δ antibodies. Results are representative of at least 3–5 separate experiments. (TIF) [file pone.0088405.s001.tif]

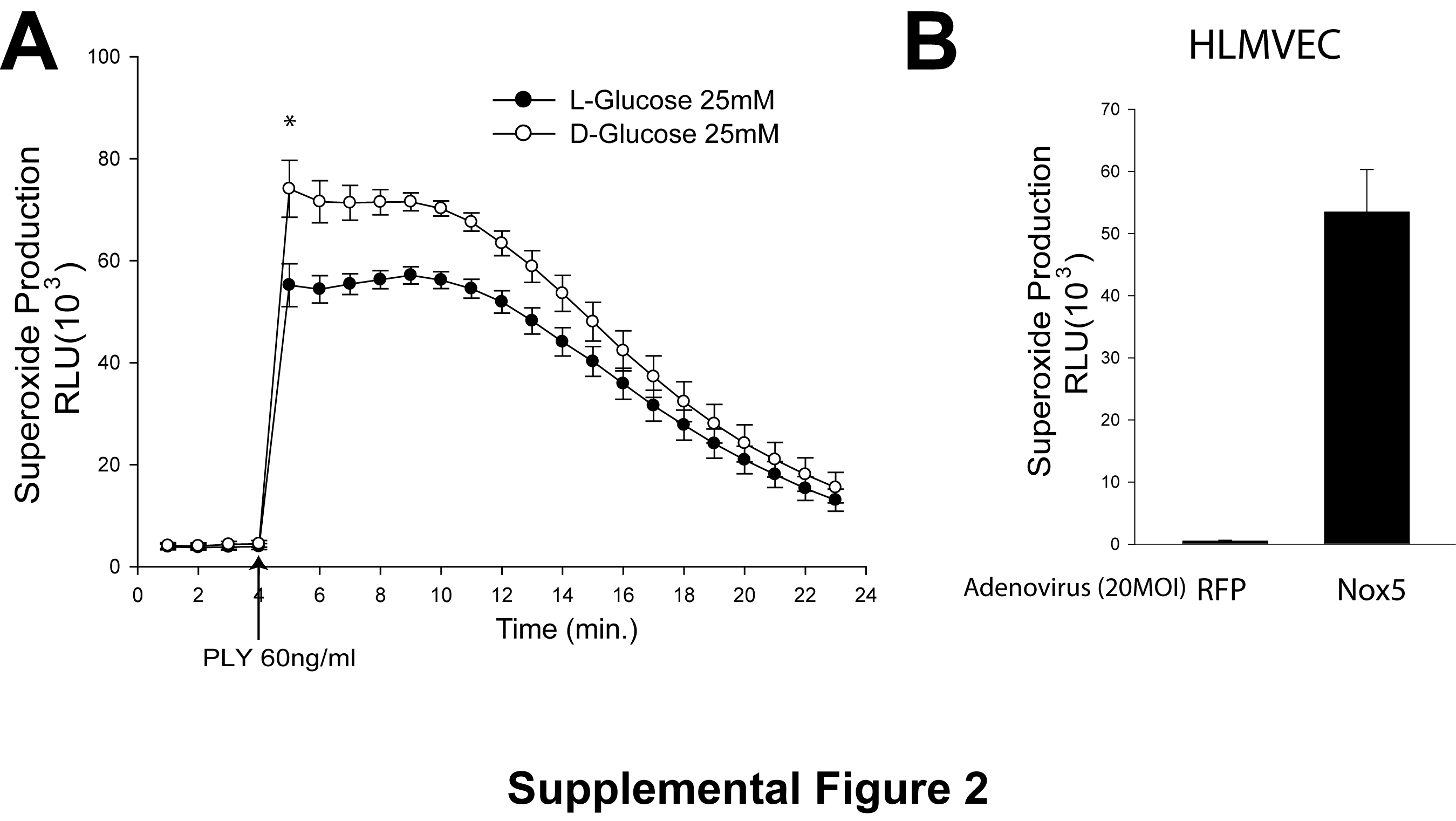

Supplement: Figure S2 — High glucose increases Nox5 derived superoxide production in HLMVEC in response to PLY. (A) HLMVC cells were infected with Nox5 adenovirus (20 MOI) for 48 hrs, and then treated with L-Glucose (25 mM) or D-glucose (25 mM) for 6 hours. Superoxide production was measured in response to PLY (60 ng/ml). Results are presented as means ± S.E., n = 6, * p<0.05 versus L-Glucose. (B) HLMVC cells were infected with RFP or Nox5 adenovirus (20 MOI) for 48 hrs, and superoxide production was measured using L-012 chemiluminescence. (TIF) [file pone.0088405.s002.tif]
